# *Bacillus velezensis* LUB-8 bioaugmentation: a strategy for lactic acid reduction in pit mud of Chinese *nong-xiang baijiu*

**DOI:** 10.1128/spectrum.03995-25

**Published:** 2026-04-30

**Authors:** Guiqiang He, Qingwei Feng, Kebu Jigu, Yanqing Wang, Lijuan Gong, Yi Ma, Jian Zhou

**Affiliations:** 1 College of Life Sciences and Agri-forestry, Southwest University of Science and Technology 91609, Mianyang, Sichuan, China; 2 Liquor Making Biotechnology and Application Key Laboratory of Sichuan Province, Sichuan University of Science and Engineering, Yibin, Sichuan, China; 3 Liquor Making Biotechnology and Intelligent Manufacturing of Key Laboratory of China National Light Industry, Yibin, Sichuan, China; South China University of Technology, Guangzhou, China

**Keywords:** *Baijiu*, lactic acid, pit mud, *Bacillus velezensis*, bioaugmentation

## Abstract

Excessive accumulation of lactic acid in pit mud (PM) is a key issue causing PM aging and affecting the quality of Chinese *nong-xiang baijiu*. This study proposes a biofortification strategy involving inoculation with *Bacillus velezensis* LUB-8 to regulate the PM microbial community and degrade lactic acid. Pure culture experiments demonstrated that the LUB-8 strain efficiently utilizes lactic acid, converting it into pyruvate and acetic acid. Lactic acid degradation reached 1.65 ± 0.04 g, with a degradation rate of 82.65%. During a 60-day PM fermentation experiment, lactic acid content in LUB-8 biofortified PM decreased significantly by 9.63% compared to the control group. High-throughput sequencing analysis revealed that bioaugmentation significantly altered the microbial community structure of the PM, increasing the relative abundance of *Caproiciproducens* and *Rummeliibacillus* and restructuring the microbial interaction network. Furthermore, PICRUSt2 functional prediction further revealed that the abundance of key enzyme-encoding genes involved in lactate utilization and acetate/butyrate synthesis pathways was significantly upregulated in the bioaugmented group. In summary, this study confirms the potential of *B. velezensis* LUB-8 in mitigating lactic acid accumulation in PM, providing an effective regulatory strategy for improving the microbial ecosystem in *nong-xiang baijiu* fermentation.

**IMPORTANCE:**

Lactic acid overload in long-term used pit mud (PM) disrupts nong-xiang baijiu fermentation, a major challenge for the *baijiu* industry. This study validates indigenous *Bacillus velezensis* LUB-8 as an effective bioaugmentation agent for lactic acid reduction. It offers a practical, eco-friendly solution to maintain PM quality by reshaping functional microbiota, avoiding harsh chemical treatments. The findings help stabilize *baijiu* production, preserve product consistency, and provide a scalable strategy for the *baijiu* industry to address PM deterioration, supporting sustainable development.

## INTRODUCTION

*Baijiu*, one of traditional fermented foods in China, is widely consumed by national and becomes more and more prevalent in East Asia, even in western countries (1). Among them, *nong-xiang baijiu* is renowned through the *baijiu* market for its deep historical heritage and unique flavor with richness of pit-aroma, soft and sweet, harmonious flavor, and long aftertaste (2). It is widely known that the typical flavor compounds are predominantly ethyl caproate in harmonious balance with ethyl lactate and other aroma contributors in Chinese *nong-xiang baijiu* (3, 4). In particular, the richness of pit-aroma in *nong-xiang baijiu* is mainly ascribed to its special brewing process of anaerobic solid-state fermentation in the mud pit (as called fermentation pit) (5). In general, the grains mainly including sorghum, corn, rice, sticky rice, and wheat are fermented about 60 days in the mud pit with Daqu starter as saccharification and fermentation agent by a recycling process (Fig. S1).

Importantly, the four walls and bottom layer of the fermentation pit are covered with a kind of specific yellow clay, also called pit mud (PM), which harbors multiple brewing microbiota for *nong-xiang baijiu* fermentation (6). Microbes inhabiting in the PM drive fermentation and produce flavor compounds mainly including acids, alcohols, and esters. For example, PM was the sustained-release source of anaerobes, which accounted for over 14% of prokaryotic communities and mainly influenced the metabolism of organic acids and flavor compounds in the fermented grain (7). Especially, the caproic acid bacteria are considered to be important functional microbiota in the PM for producing precursor of the characteristic flavor compounds (ethyl caproate) of Chinese *nong-xiang baijiu* and maintaining the stability of brewing microecosystem (8–10).

It is noteworthy that the production process of *nong-xiang baijiu* described above (Fig. S1) is periodically repeated in the same fermentation pit. The pits are generally continuously used for decades, even a century, and thus, the properties and microbiome of PM are evolved during the long-term production (11). Unfortunately, the PM may be degradated under the inappropriate brewing and maintain conditions in the production process. The essence of PM degradation is accumulation of lactic acid, which leads to the formation of calcium lactate and ferrous lactate, consequently, resulting in PM becoming light gray, dry, and hard (12, 13). Moreover, accumulation of lactic acid in PM could cause the formation of excessive ethyl lactate in *nong-xiang baijiu* and then destroy the harmonization of taste and flavor (14, 15). Therefore, it is interesting that to efficiently achieve the reduction of lactic acid content in the PM, preventing it from degradation and affecting the fermentation of Chinese *nong-xiang baijiu*.

In general, the reduction of lactic acid or ethyl lactate content in PM and *nong-xiang baijiu* is mainly by regulating the process parameters including scientific construction of the fermentation pit, optimization of the PM, and improvement of the quality of *Daqu* starters (16, 17). However, these studies focused mainly on revealing the relevances between the lactic acid/ethyl lactate contents and the controlling parameters of *nong-xiang baijiu* fermentation. The essential information involved in the succession of functional microbiota caused by these process parameters for achievement of “lactic acid or ethyl lactate reduction” is still fragmented. In recent years, the bioaugmentation approach by inoculating lactic acid-degrading bacteria in the production of *nong-xiang baijiu* is employed to effectively reduce the content of lactic acid or ethyl lactate, although the reinforcement mechanism is still not fully uncovered (18).

In the present study, *Bacillus velezensis* LUB-8, a lactic acid-degrading bacteria, was isolated from the PM of *nong-xiang baijiu*. The feasibility of the bioaugmentation method by inoculation with *B. velezensis* LUB-8 for reducing the lactic acid content in PM was evaluated. To underline how the functional strain *B. velezensis* LUB-8 influences the lactic acid metabolism, the organic acids, microbial community and interactions, and the correlations between functional microbiota and lactic acid were comprehensively analyzed in the bioaugmented PM. Furthermore, function prediction and metabolic correlation analyses were applied to improve our knowledge regarding the lactic acid reduction in PM by bioaugmentation inoculation with *B. velezensis*.

## MATERIALS AND METHODS

### Strains, growth conditions, and sample treatment

*Bacillus velezensis* LUB-8 was isolated and screened from PM in the laboratory and stored in the China Center for Type Culture Collection with serial number CCTCC NO: M 20231667. The strain was preserved at −80°C in 50% (vol/vol) glycerol solution. To obtain the original culture of the strain, *B. velezensis* LUB-8 cultivate it in a medium with lactate as the sole carbon source (LSC) at 30°C. The formula of LSC medium and the determination method of degradation performance were in the supplementary material. Measure the growth of the strain by utilizing a UV spectrophotometer (Thermo Scientific, NC2000, MA, USA). Monitor the content of organic acids in the fermentation broth using High-Performance Liquid Chromatography (HPLC) (Agilent Technologies, Palo Alto, CA, USA). After overnight fixation in a 2.5% (vol/vol) glutaraldehyde solution at 4°C, the samples were dehydrated using ethanol solutions of varying concentrations according to the method established by Zand et al. (19). The dehydrated samples were then frozen for 24 h, followed by vacuum drying, gold sputter coating, and observation using scanning electron microscopy (SEM) (FE-SEM, 200 kV, Ultra55, Carl Zeiss, Germany).

### Sample handling and sampling process

PM was taken from a distillery company in Mianyang, Sichuan Province, China. After repeated mixing of the retrieved PMs, it was evenly divided into six portions to ensure consistency in the initial state. A bacterial suspension of *B. velezensis* LUB-8 at a concentration of 2 × 10⁶ CFU/mL was prepared from the same batch and thoroughly mixed with 300 g of PM to serve as the treatment group (T). For the control group (C), an equal volume of sterile culture medium (in place of the bacterial suspension) was mixed thoroughly with 300 g of PM. Subsequently, the above-mentioned samples will be placed into sealed containers and incubated under anaerobic conditions at 30°C. After 60 days of fermentation, samples were collected using a five-point sampling method, mixed uniformly, and categorized as samples for this group. The samples were stored at −20°C until analysis.

### Simulated fermentation process for *Baijiu*

Two circular containers with a volume of 4,500 mL were coated with cultured PM to simulate the cellar environment. The PM with additional *B. velezensis* LUB-8 was used as the treatment group (T), and the PM without *B. velezensis* LUB-8 was used as the control group (C): the pre-soaked sorghum was steamed in a sterilizer for 1 h after 24 h of pre-soaking. After the sorghum was completely skin-broken, it was spread out and cooled. After the sorghum was cooled, 15% Daqu was added and stirred well. The mixture was then packed into containers, covered with PM on top, and sealed with cling film to isolate the air. Anaerobic fermentation was carried out at 30°C for 60 days. At the end of fermentation, the rice hulls will be mixed with steamed sorghum at a ratio of 0.15:1 and placed in a still for distillation, and 30 mL of the initial distillate will be discarded. The alcohol concentration of the distillate will be measured through the use of an alcohol meter, and distillation will be stopped when 50% alcohol content is reached.

### Determination of organic acids

To extract organic acids, accurately weigh 5.00 g PM and add it to the container along with 45 mL of 9.00 mM sulfuric acid (H_2_SO_4_). Organic acids were fully extracted by Zhang et al. (20) method. Collect the supernatant and purify it through a C18E column (500 mg/3 mL, Welch, Shanghai, China). Finally, the sample was filtered through an injector filter (0.22 μm, JinTeng, Tianjin, China) for use in HPLC analysis.

Utilizing the Agilent 1260 HPLC system (Agilent Technologies, Palo Alto, CA), equipped with a variable wavelength UV/visible detector and an Alltech OA-1000 organic acid column (300 mm × 6.5 mm, GRACE, Columbia, USA), for the HPLC analysis of organic acids. The collection and analysis of chromatographic data will be carried out using the Agilent Chemstation software. The mobile phase consisted of 9.00 mM H_2_SO_4_, with a flow rate of 0.60 mL/min, 15 μL per injection. The oven temperature was set at 65°C, and the detection wavelength was 210 nm (21). Using five different concentrations of organic acid standards including lactic acid, acetic acid, pyruvic acid, butyric acid, and caproic acid for HPLC analysis, a calibration curve was established (Table S1), and the content of each organic acid was calculated.

### Determination of flavor substances in crude baijiu

The contents of four major acids and esters in *baijiu* were determined by gas chromatography (22). The chromatographic column was a 60 m × 250 μm × 0.25 µm HP-INNOWax column with an inlet temperature of 240°C, the carrier gas was 99.999% high-purity helium at a flow rate of 1 mL/min, the injection volume was 1 μL, the split ratio was 20:1, the initial temperature was maintained at 40°C for 5 min, and then the temperature was elevated to 230°C at 4°C/min, and then elevated to 250°C at 10°C/min for 3 min. The lactic acid content in the crude baijiu sample was measured by HPLC method as described in the Determination of organic acids section; contents of acetic acid, butyric acid, caproic acid, ethyl lactate, ethyl acetate, ethyl butyrate, and ethyl caproic acid in the crude baijiu sample were detected by gas chromatography; the above substances were quantified using the internal standard method; and the standard curves were plotted (Table S2).

### DNA extraction, PCR amplification, and sequencing analysis

After processing an appropriate amount of PM using a tissue grinder (Tissuelyser-48, Shanghai, China), DNA extraction from the PM was carried out using the OMEGA Soil DNA Kit (D5635-02) (Omega Bio-Tek, Norcross, GA, USA) as per the manufacturer’s instructions. DNA molecules were assessed for size using 0.8% agarose gel electrophoresis and quantified using Nanodrop (Thermo Scientific, NC2000, MA, USA). To amplify the bacterial 16S rRNA V3-V4 region, specific primers 338F (5′-ACTCCTACGGGAGGCAGCA-3′) and 806R (5′-GGACTACHVGGGTWTCTAAT-3′) were employed. The PCR parameters were set according to the previously established protocol He et al. (23). The PCR amplification results were examined using 2% agarose gel electrophoresis, and the target fragments were excised and recovered using the AxyPrep DNA Gel Extraction Kit (AP-GX-250, Axygen, USA). The sequencing library was prepared using the TruSeq Nano DNA LT Library Prep Kit. For qualified libraries, dual-end sequencing with 2 × 250 bp was conducted on the Illumina NovaSeq platform using the NovaSeq 6000 SP Reagent Kit (500 cycles, Personal, China).

Microbiome bioinformatics analysis was performed using the QIIME2 software (24). The raw sequence data were decoded and processed using the demux plugin. The cutadapt plugin was used for primer trimming, followed by the implementation of the DADA2 plugin for quality filtering, denoising, merging, and chimera removal of the sequences. The obtained sequences were merged based on 100% sequence similarity, resulting in the generation of feature-specific sequences called Amplicon Sequence Variants (ASVs) and abundance data table (25). The ASV feature sequences were compared to the reference sequences in the Greengenes database to obtain taxonomic information corresponding to each ASV (26).

### Data analysis

The alpha diversity index of the PM was calculated using the ASVs. Co-occurrence analysis of microbial communities was conducted by calculating the Spearman’s rank correlation coefficient (RHO), with significant microbial interaction networks established under the conditions of |*R*| > 0.6 and *P* < 0.01. Significant differences in organic acid concentrations among PM samples were analyzed by one-way analysis of variance (ANOVA), and each treatment consisted of three biological replicates (*n* = 3). Redundancy analysis (RDA) was employed to uncover the correlations between microbial communities and organic acids within different groups. Based on the KEGG PATHWAY database, potential functions related to organic acid metabolism in PM microbial communities were predicted using PICRUSt2 (27).

## RESULTS AND DISCUSSION

### Metabolic characteristics of *B. velezensis* LUB-8

**Fig 1:**
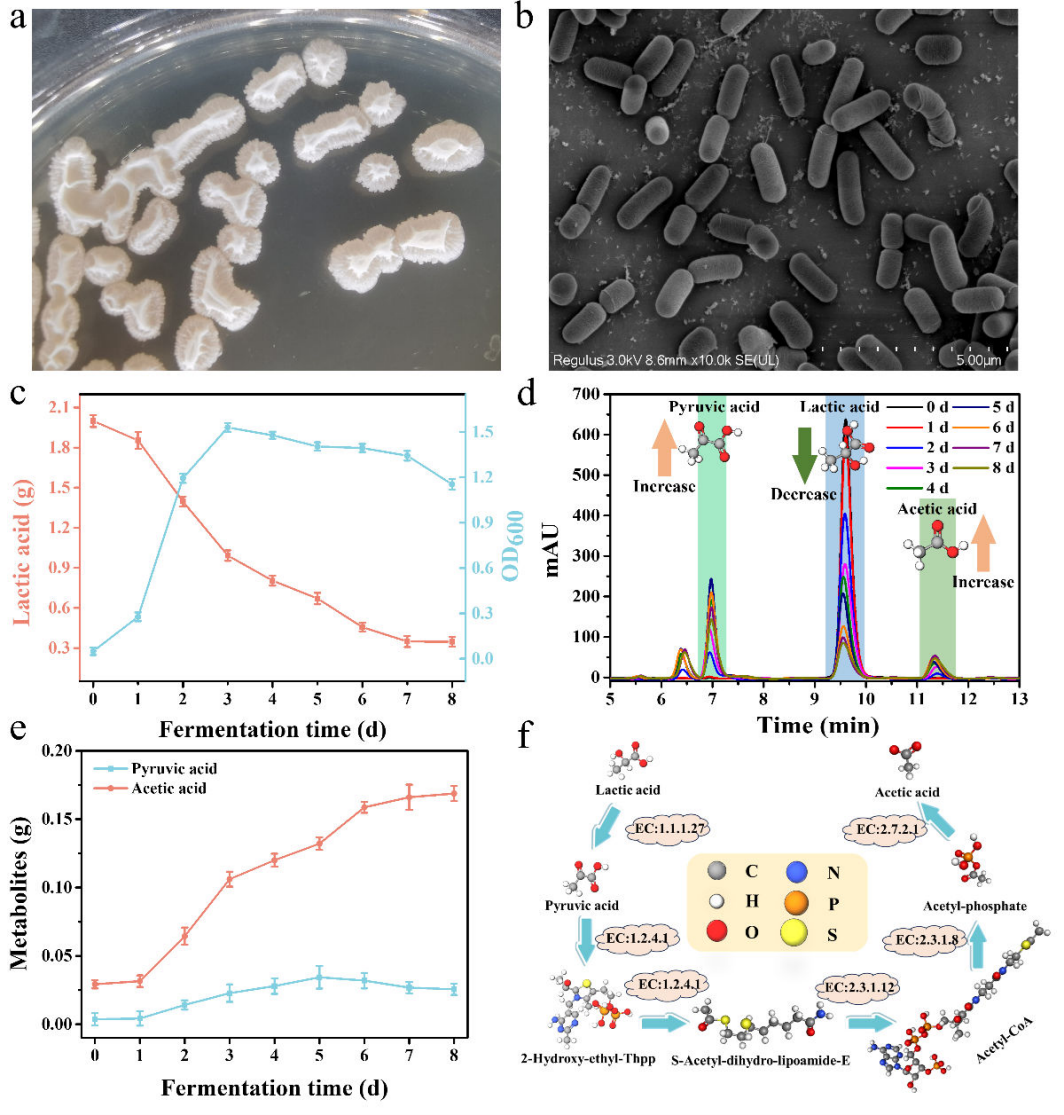
Morphological and metabolic characteristics of *B. velezensis* LUB-8. (**a**) Colony morphology on LSC medium; (**b**) SEM image; (**c**) growth characteristics and lactic acid degradation curve of *B. velezensis* LUB-8; (**d**) HPLC chromatogram of fermentation; (**e**) change of metabolite content of strain; (**f**) predicted metabolic pathway.

After preliminary screening, lactate-degrading bacteria were successfully isolated from PM. Through sequence alignment, this strain was identified as *B. velezensis* (Fig. S3). The strain appeared as white, irregular, and exhibited wrinkled surfaces with slight stickiness (Fig. 1a). Under SEM observation, the strain was found to have a short, rod-shaped structure (Fig. 1b). It was observed that after fermentation for 8 days, it could effectively degrade 1.65 g of lactic acid, resulting in a lactic acid degradation rate of 82.65% (Fig. 1c). From Fig. 1d, it can be observed that as lactic acid degrades, the levels of pyruvic acid and acetic acid gradually increase. The level of pyruvic acid gradually increases to 0.03 g and then stabilizes. At the same time, the level of acetic acid gradually increases, and by the 8th day, the fermentation broth has produced 0.17 g of acetic acid (Fig. 1d and e). This phenomenon is highly likely to be caused by the degradation of lactic acid (28). As reported, the metabolic byproducts of lactate-degrading bacteria also include organic compounds such as aldehydes, alcohols, and esters (29). The production of these substances may contribute to the accumulation of ethyl caproate in *nong-xiang baijiu*, enriching the flavor components.

The high versatility of *Bacillus velezensis* has garnered significant attention in the scientific community in recent years (30), making its biosafety a prerequisite for industrial application. Regarding the practical value of the species, *B. velezensis* has been widely applied in the biofortification of Daqu (31, 32). In the specific context of this study, strain LUB-8 was employed for the acclimation of PM during the solid-state anaerobic fermentation of *baijiu*. Rather than directly participating in the formation of the liquor body, the strain functions by regulating the micro-ecological balance within the PM. Furthermore, the high-temperature distillation process inherent to *baijiu* production serves as a critical barrier, eliminating residual microorganisms in the base liquor and effectively mitigating the potential risk of bacterial residue in the final product. Additionally, the metabolites of LUB-8 within the PM system primarily consist of small-molecule organic acids and enzymes associated with lactate degradation, and the absence of toxic or harmful secondary metabolites further ensures the safety of its application in *baijiu* fermentation.

### Effect of bioaugmentation on organic acids in PM and crude *baijiu*

**Fig 2:**
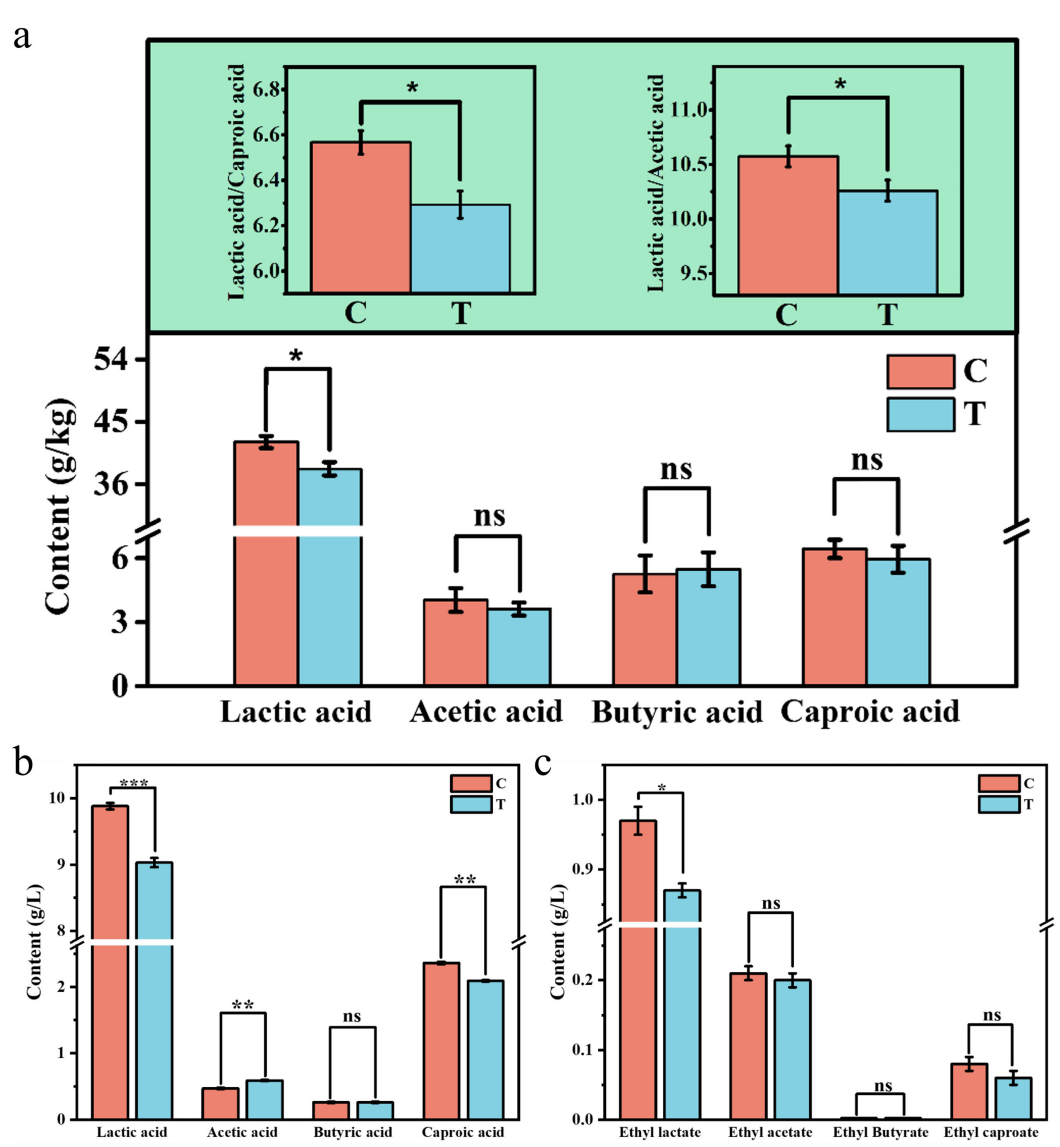
Changes of organic acid content. (**a**) Organic acid content in PM; (**b**) flavor compound content in crude *baijiu* (e.g., *P < 0.05; **P < 0.01; ***P < 0.001; ns, not significant).

In order to investigate the impact of *B. velezensis* LUB-8 sporulation disturbance on the organic acid content in PM, two sets of samples were tested for organic acid in the PM after anaerobic fermentation for 60 days. The results indicated that the lactic acid content of the treatment group was significantly decreased from 42.07 g/kg to 38.20 g/kg (*P* < 0.05). Although there were varying fluctuations in the levels of acetic acid, butyric acid, and caproic acid compared to the control group, no significant differences were observed. In addition, the ratio of lactic acid to caproic acid and lactic acid to acetic acid in PM decreased significantly (*P* < 0.05), from 6.56 to 6.29 and from 10.57 to 10.26, respectively (Fig. 2). This indicates that *B. velezensis* LUB-8 can effectively balance the proportion of acids and reduce lactic acid in PM. This is most likely due to the effect of lactate dehydrogenase, which promotes the conversion of lactic acid into other substances. Lactic acid, primarily produced by *Lactobacillus* metabolism, plays a positive role in regulating the pH of the brewing microecology and inhibiting the growth of unwanted bacteria (33). However, an excessive amount of lactic acid not only intensifies degradation in PM but also leads to a deterioration in the taste and texture of the *baijiu*.

The main flavor compounds in crude *baijiu* distilled from each group were detected by gas chromatography and liquid chromatography. The results are shown in Fig. 2b and c. Compared with the control group, the content of lactic acid and ethyl lactate in the treated group was significantly reduced, lactic acid decreased from 9.88 g/L to 9.03 g/L, a decrease of 8.60%, while the content of ethyl lactate decreased from 0.97 g/L to 0.87 g/L, a decrease of 10.31%. The content of ethyl lactate in the crude *baijiu* was effectively reduced, which suggests a potential positive regulatory effect on the flavor substance profile of the crude *baijiu* and provides a preliminary indication of improved flavor balance in the product. In addition, *B. velezensis* LUB-8 reduced the content of lactic acid in the fermentation system by converting acetic acid. This process was accompanied by the synchronous increase of acetic acid content in the wine. As a key precursor for the synthesis of butyric acid and caproic acid, the increase of acetic acid content could provide a more sufficient substrate for the subsequent accumulation of butyric acid and caproic acid and indirectly promote the production of target flavor organic acids.

### Effects of bioaugmentation on bacterial community diversity

After quality filtering, denoising, merging, and removing chimeric sequences, high-quality sequences in PM were obtained. The total number of valid sequences obtained from the control group and treatment group were 97,553–104,361 reads and 96,029–102,421 reads. The number of high-quality sequences being 68,320–80,536 reads and 66,123–76,626 reads, respectively (Table S3). Furthermore, the observed species richness curve gradually stabilizes (Fig. S3), indicating that the sequencing results accurately reflect the microbial diversity in PM.

**Fig 3:**
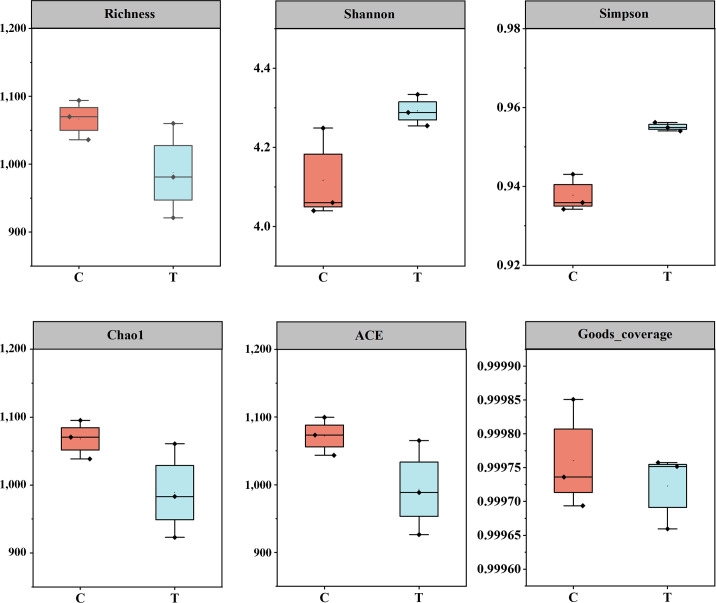
Alpha diversity difference of the microbial community index among samples.

Moreover, based on the effective reads, 2,169 ASVs and 2,036 ASVs were, respectively, clustered in different groups, with a similarity threshold of 100%. Next, the α-diversity indices of bacterial communities in the two groups were calculated in detail based on the clustered ASVs. As shown in Fig. 3, bacterial abundance decreased after bioaugmentation. Furthermore, the Shannon index and Simpson index were evaluated. It can be observed that the use of *B. velezensis* LUB-8 significantly increased the species diversity and evenness in the PM. The Chao1 index and the ACE index represent the total number of species in the PM. It indicates that the use of *B. velezensis* LUB-8 reduces certain species. Additionally, the decrease in richness index also confirms this point. These results were consistent with reference 32. These findings can be attributed to the complex microbial interactions that impact community structure and functionality.

### Effects of bioaugmentation on bacterial community structure

**Fig 4:**
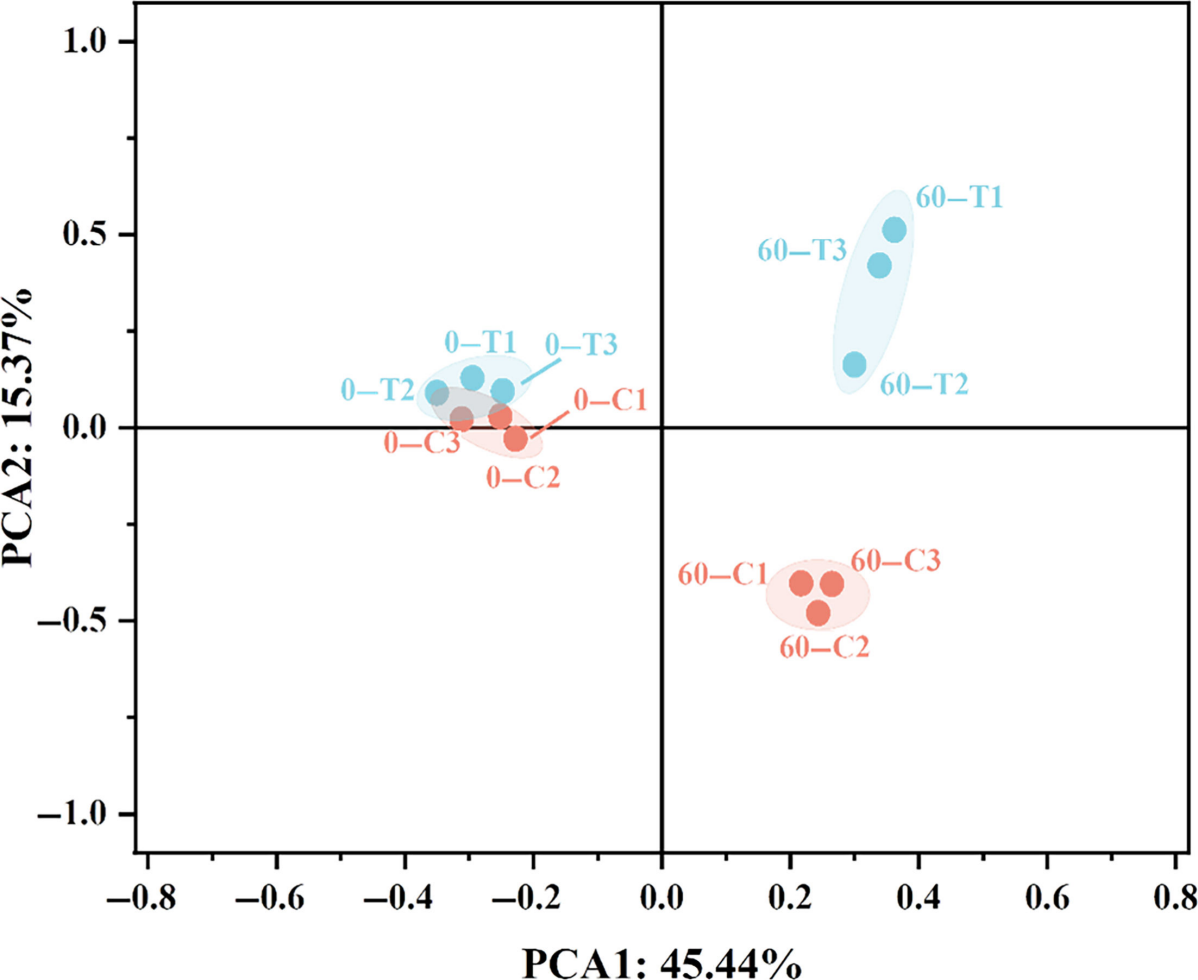
Results of PCA sequencing of PM.

Based on the principal component analysis (PCA) of the bacterial community using the detected ASVs in the samples, as shown in Fig. 4. The initial state of PM (0C and 0T) remained the same, but the microbial community structure changed greatly after fermentation. These two axes account for 60.81% of the total variation in the bacterial community. The fermented samples were classified into two distinct clusters, with the treatment group separated from the control group along the PCA2 axis. This suggests that the addition of *B. velezensis* LUB-8, indeed, has an impact on the microbial community structure in the PM. To further elucidate the changes in microbial community structure, the microbiome composition at phyla and genus levels in PM was analyzed.

**Fig 5:**
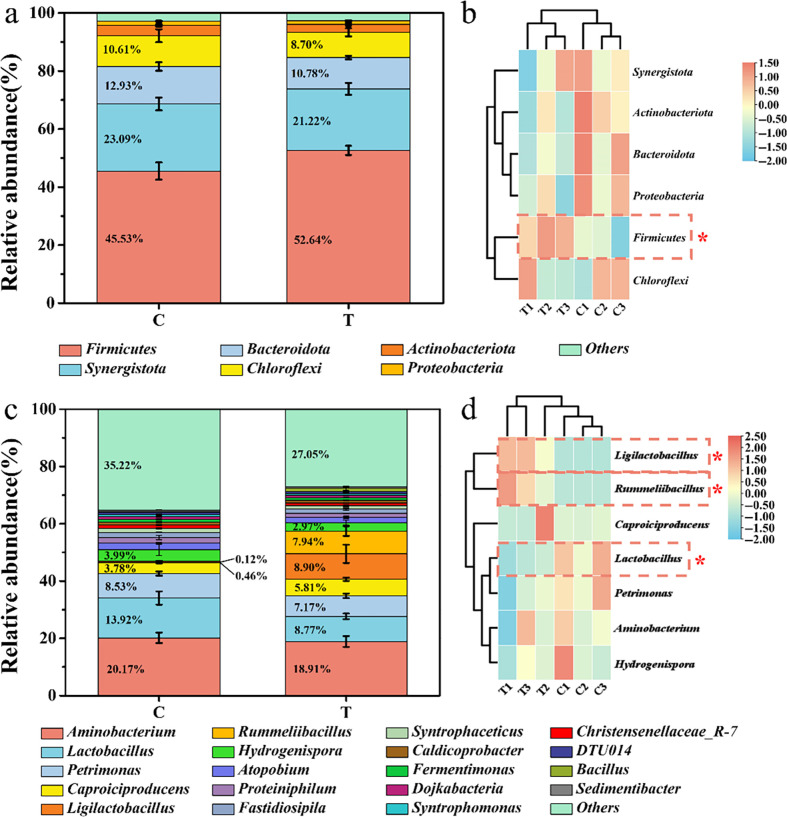
Microbial community composition of PM samples at phylum level (**a and b**) and genus level (**c and d**). * indicates significant difference (*P* < 0.05).

At the phylum level, the PM was mainly composed of *Firmicutes*, *Synergistota*, *Bacteroidota*, and *Chloroflexi*, which were frequently detected as dominant bacterial phyla during the fermentation process of *baijiu* (34). In addition, it can be observed that after intensifies, the abundance of the *Firmicutes* significantly increased from 45.53% to 52.64% (*P* < 0.05) (Fig. 5). *Firmicutes* plays a crucial role in the fermentation process of traditional *baijiu* due to its ability to synthesize short-chain fatty acids (35). The increased abundance of *Firmicutes* assisted in the production of flavor substances during the fermentation in *baijiu*. Moreover, through the cluster heatmap analysis (Fig. 5b), the six samples were divided into two clusters. Interestingly, the samples from the treatment group were well differentiated from the control group.

At the genus level, the analysis was conducted on the top 19 abundant genus, while the remaining genus were categorized as “Others.” As shown in Fig. 5c and d, the PM predominantly consisted of seven genera, namely, *Aminobacterium*, *Lactobacillus*, *Petrimonas*, *Caproiciproducens*, *Ligilactobacillus*, *Rummeliibacillus*, and *Hydrogenispora* (Fig. 5c). Compared to the control group, the abundance of *Aminobacterium* in the treatment group decreased from 20.17% to 18.91%. The abundance of *Lactobacillus* significantly decreased from 13.92% to 8.77% (*P* < 0.05), while the content of *Caproiciproducens* increased from 3.78% to 5.81%. Additionally, the abundance of *Ligilactobacillus* and *Rummeliibacillus* significantly increased (*P* < 0.05) from 0.46% to 8.9% and from 0.12% to 7.94%, respectively (Fig. 5d). Bidirectional regulation of microbiota was achieved by the addition of *B. velezensis* LUB-8. Furthermore, *B. velezensis* LUB-8 utilizes lactic acid as a metabolic substrate, which helps alleviate acid stress in the fermentation environment, leading to the growth of acid-sensitive microorganisms. According to the report, *Bacillus licheniformis* was defined as an effective regulator of microbial community structure and function in Chinese *baijiu* fermentation. When *B. licheniformis* was inoculated in *Daqu*, the abundance of *Lactobacillus* decreased significantly (36). This finding is consistent with our research results, which also demonstrate the effective conversion of lactic acid and *Lactobacillus* abundance in the fermentation environment. Therefore, *Bacillus* makes unique contributions to microbial enrichment, flavor production, and PM maintenance.

### Effects of bioaugmentation on interspecies interactions of microorganisms

**Fig 6:**
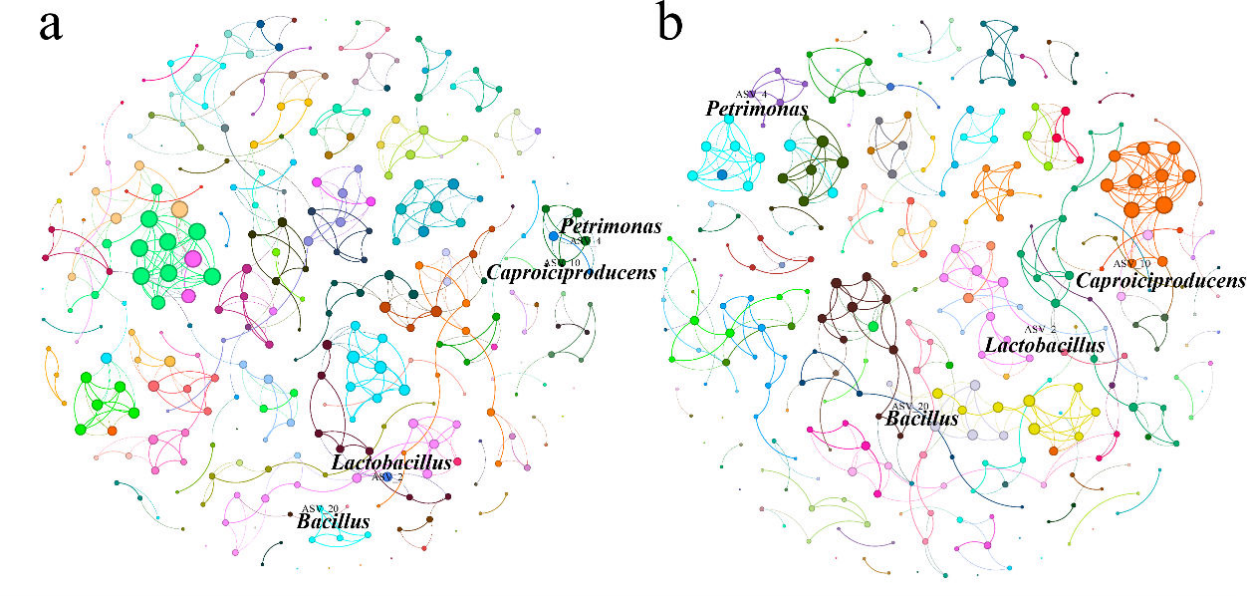
Correlation analysis between control group (**a**) and treatment group (**b**) microbial communities. Each circle represents a microbe, and the lines represent the degree of correlation between elements (*P* < 0.01; Spearman’s rho > 0.6). Different colored circles correspond to different clusters of elements.

To investigate whether *B. velezensis* LUB-8 affects the interactions among microbial in the PM, microbial interaction networks for the control and treatment groups were plotted (Fig. 6). It is evident that there have been changes in the interactions among microorganisms in the PM. For example, before bioaugmentation, there was a correlation between the microorganisms *Caproiciproducens* and *Petrimonas* in the PM. However, after bioaugmentation, *Caproiciproducens* showed more complex associations with other organisms. Notably, the link between the bacillus and other microorganisms was also enhanced, such as *Petrimonas*, *Clostridium_sensu_stricto_3,* and *Arenimonas*. It has been reported that *Petrimonas* and *Clostridium_sensu_stricto_3* were not only dominant species in the PM but also directly associated with the accumulation of propionic acid and caproic acid (37). Therefore, *B. velezensis* LUB-8 bioaugmentation leads to a change in the structure of microbial ecosystem network in PM and a shift in the direction of propionic acid and hexanoic acid accumulation.

### Microbial community assembly in biofortified PMs

**Fig 7:**
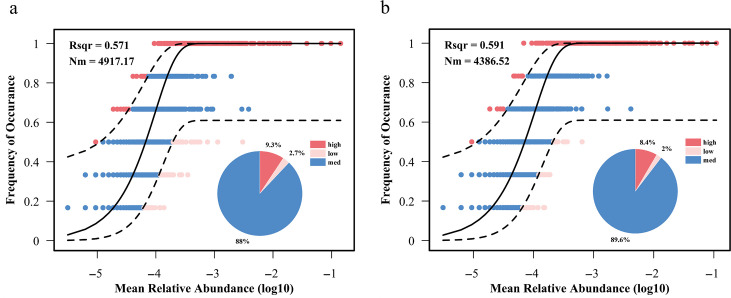
Microbial community construction of PM in the control group (**a**) and treatment group (**b**). The red and light pink circles indicate the ASVs whose actual occurrence frequencies are higher and lower, respectively, than the predicted value. The black dotted line represents the 95% confidence interval of the predicted value of the model, and the circle within this interval is regarded as the neutral distribution. The pie chart shows the relative abundance distribution of higher than predicted, lower than predicted, and neutral distribution.

Neutral Community Model (NCM), proposed by Sloan et al. (38), is a classical method to quantify the contribution of stochastic processes to community assembly and to resolve the correlation between the relative abundance and frequency of occurrence of taxonomic units in the community and has been widely used in the study of prokaryotic microbial community building mechanisms. To clarify the role of the *B. velezensis* LUB-8 domestication process in regulating the assembly pattern of microbial communities in PM, the present study was based on NCM analysis of the relationship between frequency of occurrence and relative abundance of bacterial ASVs in the experimental and control groups, to reveal the differences in the core-driven processes of community assembly between the two groups (Fig. 7). The results showed that stochastic processes contributed slightly more to the experimental group than the control group, explaining 59.1% and 57.1% of the community variation, respectively. Mobility (*m*) indicates the ability of species to spread at the community level (39). The higher the value of *m*, the easier it is for the species to migrate and the less restricted the spreading is. The migration rate of the experimental group was slightly lower than that of the control group, which suggested that the diffusion process of microbial species in the PM of the experimental group was more restricted. These results suggest that the assembly of microbial community in PM is more likely to be driven by stochastic processes after intensive treatment with *B. velezensis* LUB-8, which may be caused by the following mechanisms: first, the introduction of *B. velezensis* LUB-8 broke the original microecological balance of the PM, and its competitive and symbiotic interactions with microorganisms of the original PM remodeled the network of inter-species associations and interfered with the deterministic associations. *B. velezensis* LUB-8 could reduce the lactic acid content in the PM, and the decrease in lactic acid concentration altered the selection pressure in the PM microenvironment, weakened the directional screening effect of deterministic processes on microbial growth, and indirectly enhanced the contribution of stochastic processes in the community assembly.

### Correlation between microbiota and organic acids

**Fig 8:**
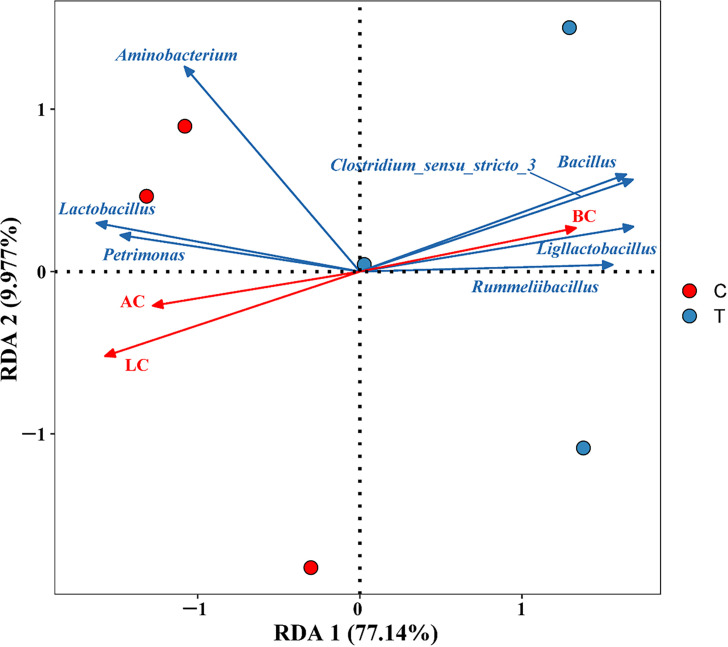
The RDA results of dominant microorganisms and organic acids. AC, acetic acid; LC, lactic acid; BC, butyric acid.

In addition, redundancy analysis (RDA) revealed the correlation between microbial communities and organic acids in the PM. The results showed a negative correlation between *Bacillus*, *Clostridium_sensu_stricto_3*, *Ligilactobacillus*, *Rummeliibacillus*, and lactic acid, while a positive correlation was observed with butyric acid (Fig. 8). It means that *Bacillus* contributed to the conversion of lactic acid in PM. Furthermore, a negative correlation between *Bacillus* and *Lactobacillus* was also observed, which is consistent with previous reports (32). Specifically, there exists a fierce competitive relationship between *Bacillus* and *Lactobacillus*, as frequently reported in previous studies. *Lactobacillus* inhibits the growth of *Bacillus* and other microorganisms in the fermentation system through pH conversion, accumulation of lactic acid, or production of bacteriocins (40).

### Analysis of functional composition of microbial community in PM

**Fig 9:**
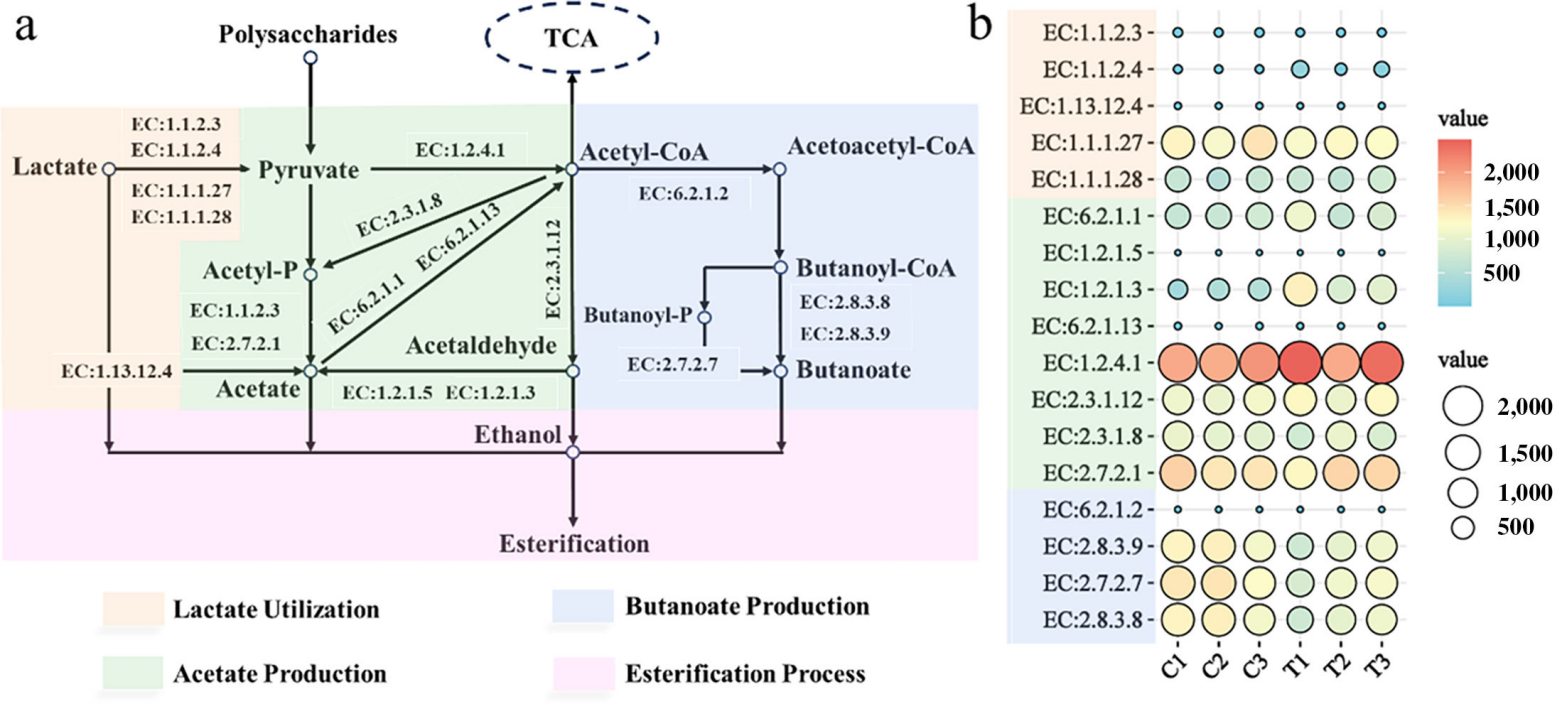
Abundance changes of functional genes encoding metabolic enzymes involved in lactate utilization and carboxylic acid chain extension were analyzed by PICRUSt2. (**a**) Lactate utilization and carboxylic acid chain extension pathway map; (**b**) the abundance of key enzymes changed. EC:1.1.2.3, L-lactate dehydrogenase (cytochrome); EC:1.1.2.4, D-lactate dehydrogenase (cytochrome); EC:1.13.12.4, Lactate 2-monooxygenase; EC:1.1.1.27, L-lactate dehydrogenase; EC:1.1.1.28, D-lactate dehydrogenase; EC:6.2.1.1, Acetate-CoA ligase; EC:1.2.1.5, Aldehyde dehydrogenase (NAD(P)(+)); EC:1.2.1.3, Aldehyde dehydrogenase (NAD(+)); EC:6.2.1.13, Acetate-CoA ligase (ADP-forming); EC:1.2.4.1, Pyruvate dehydrogenase (acetyl-transferring); EC:2.3.1.12, Dihydrolipoyllysine-residue acetyltransferase; EC:2.3.1.8, Phosphate acetyltransferase; EC:2.7.2.1, Acetate kinase; EC:6.2.1.2, Butyrate-CoA ligase; EC:2.8.3.9, Butyrate-acetoacetate CoA-transferase; EC:2.7.2.7, Butyrate kinase; EC:2.8.3.8, Acetate CoA-transferase.

Using the PICRUSt2 tool and 16S rRNA gene sequencing results, the functional profiles of bacterial communities involved in organic acid metabolism in the PM before and after *B. velezensis* LUB-8 enhancement were drawn (Fig. 9). Seventeen key enzymes involved in the utilization and production of acetate, lactate, and butanoate were focused. Specifically, enzymes related to lactate utilization (EC:1.1.2.4) and acetate production (EC:1.2.1.3) were significantly upregulated, indicating an enhanced process of lactate utilization and acetate production in the PM (*P* < 0.05). Furthermore, there was an increase in the abundance of enzymes (EC:1.2.4.1 and EC:2.3.1.12) related to pyruvic acid metabolism. This finding aligns with our previous predictions (Fig. 1f). Under the catalysis of EC:1.1.1.27, lactic acid is converted to pyruvic acid. Simultaneously, the generated electrons undergo oxidative phosphorylation through the electron transport chain, providing energy for microorganisms (41). Pyruvic acid is converted to acetaldehyde through the action of dihydrolipoyl dehydrogenase (EC:1.2.4.1) and dihydrolipoyllysine-residue acetyltransferase (EC:2.3.1.12). Acetyl-CoA undergoes transformations mediated by EC:2.3.1.8 and EC:2.7.2.1, leading to the production of acetic acid, followed by carbon chain elongation pathways. In summary, bioaugmentation by *B. velezensis* LUB-8 did accelerate the utilization of lactic acid in the fermentation system.

### Conclusion

This study confirms that *Bacillus velezensis* LUB-8 can significantly reduce lactic acid content in PM through bioaugmentation, promote its metabolic conversion, and optimize bacterial community structure and gene expression related to organic acid metabolism, thereby achieving effective regulation of the microbial ecosystem in PM for *nong-xiang baijiu*. However, this study employed only amplicon sequencing to analyze community structure and was conducted at a laboratory scale. Furthermore, the molecular mechanisms underlying the strain’s lactic acid degradation and its efficacy in industrial applications require further investigation and validation. Nevertheless, these findings provide novel functional microbial resources for regulating lactic acid accumulation in PM, offering significant reference value for applied research in brewing microbiology.

## Data Availability

All sequencing data have been deposited at the Sequence Read Archive of the National Center for Biotechnology Information, and the accession number is PRJNA1062691. Bio-Sample accessions of bacterial sequences are SAMN39308441 (C1), SAMN39308442 (C2), SAMN39308443 (C3), SAMN39308444 (T1), SAMN39308445 (T2), and SAMN39308446 (T3).

## SUPPLEMENTAL MATERIAL

Supplemental material spectrum.03995-25-s0001.docx Tables S1 to S3; Fig. S1 to S3.

ASM does not own the copyrights to Supplemental Material that may be linked to, or accessed through, an article. The authors have granted ASM a non-exclusive, world-wide license to publish the Supplemental Material files. Please contact the corresponding author directly for reuse.
