# Supplementary material for: Bacillus velezensis LUB-8 bioaugmentation: a strategy for lactic acid reduction in pit mud of Chinese nong-xiang baijiu
Source: Microbiol Spectr. 2026 Apr 30;14(6):e03995-25. doi: 10.1128/spectrum.03995-25 (PMC13228017; doi:10.1128/spectrum.03995-25)

**Supplementary materials for:**

***Bacillus velezensis* LUB-8 bioaugmentation: a strategy for lactic acid reduction in pit mud of Chinese *nong-xiang baijiu***

Guiqiang He^1,#,*^, Qingwei Feng^1,#^, Kebu Jigu^1^, Yanqing Wang^1^, Lijuan Gong^2,3^, Yi Ma^2,3,*^, Jian Zhou^1,*^

^1^ College of Life Sciences and Agri-forestry, Southwest University of Science and Technology, Mianyang, Sichuan 621010, China;

^2^ Liquor Making Biotechnology and Application Key Laboratory of Sichuan Province, Sichuan University of Science and Engineering, Yibin, Sichuan 644000, China;

^3^ Liquor Making Biotechnology and Intelligent Manufacturing of Key Laboratory of China National Light Industry, Yibin, Sichuan, 644000, China.

^#^ Both authors are co-first authors of the article.

**Running title:** Lactic acid degradation by *Bacillus velezensis*.

* **Corresponding author:**

Guiqiang He

Mailing address: College of Life Sciences and Agri-forestry, Southwest University of Science and Technology, Mianyang, Sichuan 621010, China.

Email: [guiqianghe@swust.edu.cn](mailto:guiqianghe@swust.edu.cn)

Jian Zhou

Mailing address: College of Life Sciences and Agri-forestry, Southwest University of Science and Technology, Mianyang, Sichuan 621010, China.

E-mail: [zhoujian@swust.edu.cn](mailto:zhoujian@swust.edu.cn)

Yi Ma,

Mailing address: Sichuan University of Science and Engineering, Yibin, Sichuan 644000, China.

Email: [zhcngyer2008@suse.edu.cn](mailto:zhangyer2008@suse.edu.cn)

**Formula of LSC medium:** sodium lactate 20 g/L, potassium dihydrogen phosphate 0.3 g/L, yeast powder 5 g/L, disodium hydrogen phosphate 1 g/L, sodium chloride 0.5 g/L, ammonium sulfate 5 g/L, magnesium sulfate 0.5 g/L, pH adjusted to 6.8.

**Degradation performance of *B. velezensis* LUB-8:** *B. velezensis* LUB-8 was activated in LSC medium for 48 h, diluted with sterile water to OD_600_ = 1, inoculated into 100ml LSC medium with a concentration of 20 g/L at a 1% ratio, and cultured at 30 °C. Samples were taken every 24 h, and their biomass was measured by ultraviolet spectrophotometry (Thermo Scientific, NC2000, MA, USA). The content of organic acids in fermentation broth was determined by high performance liquid chromatography (Agilent Technologies, Palo Alto, CA).

**Table S1** Standard curve for determination of organic acids based on HPLC

|  | Standard equation | R^2^ |
| --- | --- | --- |
| Pyruvic acid | y = 9099.3x -316.60 | 0.9991 |
| Lactic acid | y = 436.95x-8.14 | 0.9999 |
| Acetic acid | y = 708.68x-207.11 | 0.9994 |
| Butyric acid | y = 686.43x-5.96 | 0.9995 |
| Caproic acid | y = 576.81x-31.89 | 0.9997 |

**Table S2** Standard curve for determination of organic acids based on GC

|  | Standard equation | R^2^ |
| --- | --- | --- |
| Acetic acid | y = 0.865x-0.30 | 0.9951 |
| Butyric acid | y = 1.211x-0.62 | 0.9919 |
| Caproic acid | y = 0.598x-0.32 | 0.9952 |
| Ethyl lactate | y = 0.700x-0.05 | 0.9951 |
| Ethyl acetate | y = 0.758x-0.23 | 0.9961 |
| Ethyl butyrate | y = 1.538x+0.00 | 0.9997 |
| Ethyl caproate | y = 1.363x-0.22 | 0.9959 |

**Table S3** ASVs distribution on difference level of different samples.

| Samples | Input | Filtered | Denoised | Merged | Non-chimeric | Non-singleton |
| --- | --- | --- | --- | --- | --- | --- |
| C | 118124 | 107292 | 104361 | 92329 | 78459 | 78363 |
|  | 109793 | 100836 | 97553 | 84939 | 68320 | 68234 |
|  | 114962 | 105483 | 102847 | 93012 | 80536 | 80452 |
| T | 107918 | 98648 | 96029 | 85532 | 70626 | 70520 |
|  | 110815 | 101052 | 98798 | 89044 | 76626 | 76550 |
|  | 115048 | 105518 | 102421 | 87045 | 66123 | 65973 |

**Fig. S1**

Production process diagram of Chinese *nong-xiang baijiu.*


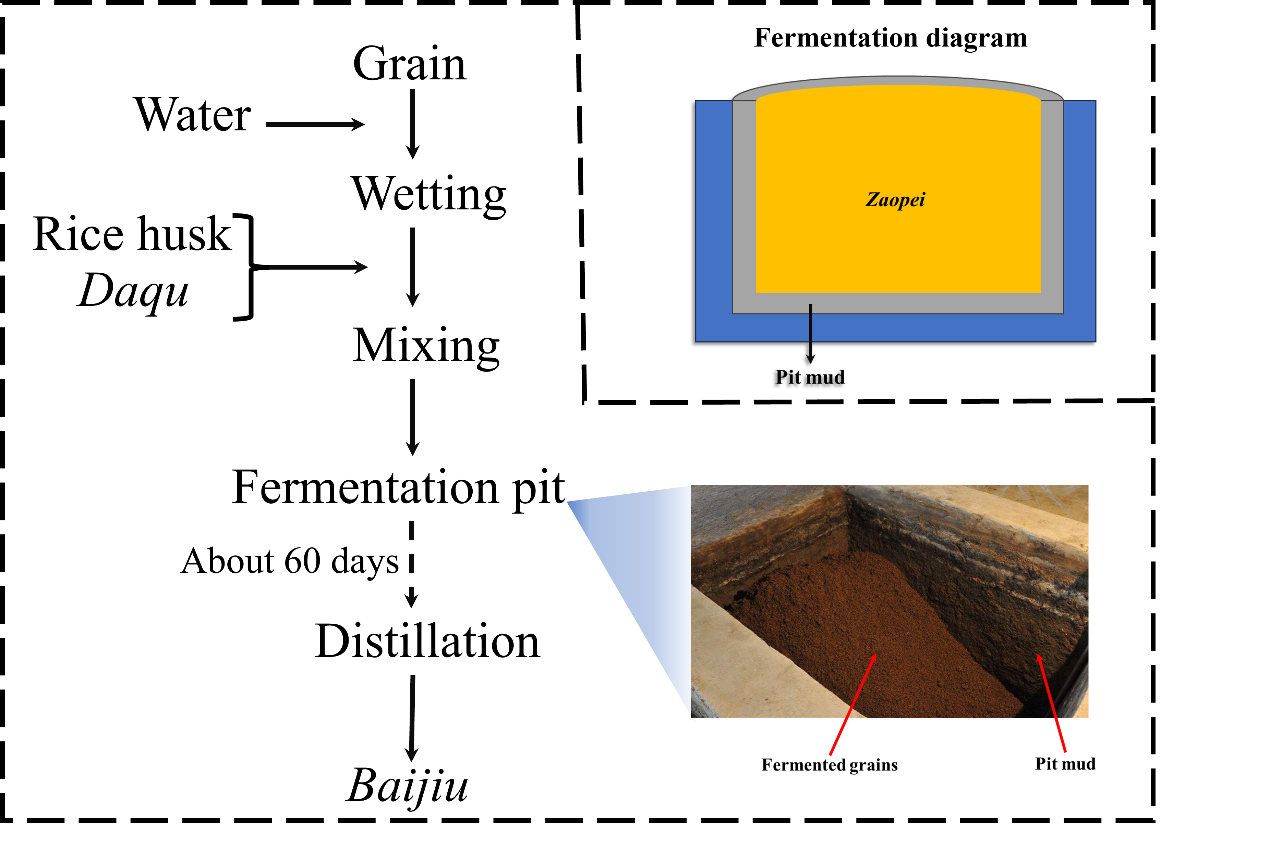


**Fig. S2**

Phylogenetic tree of lactic acid degrading bacteria.


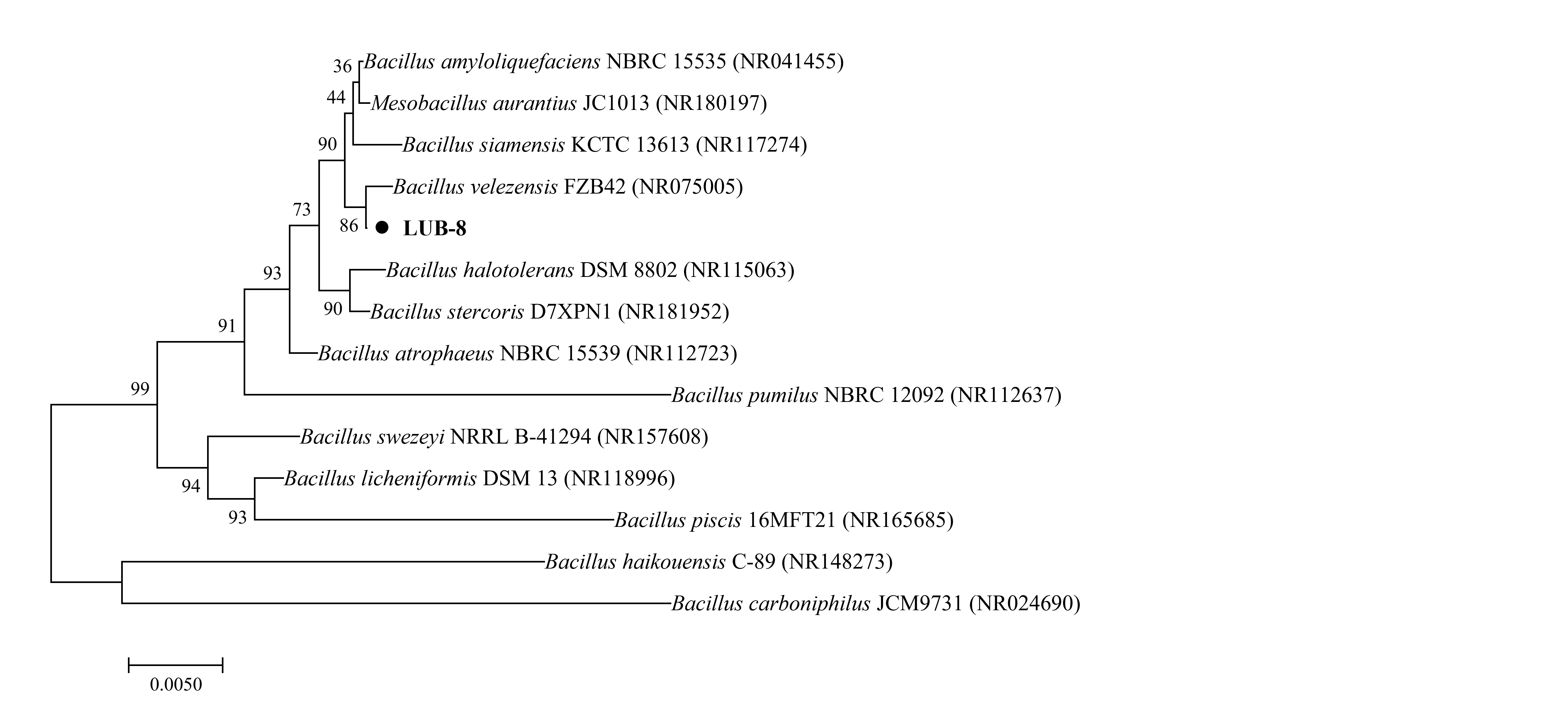


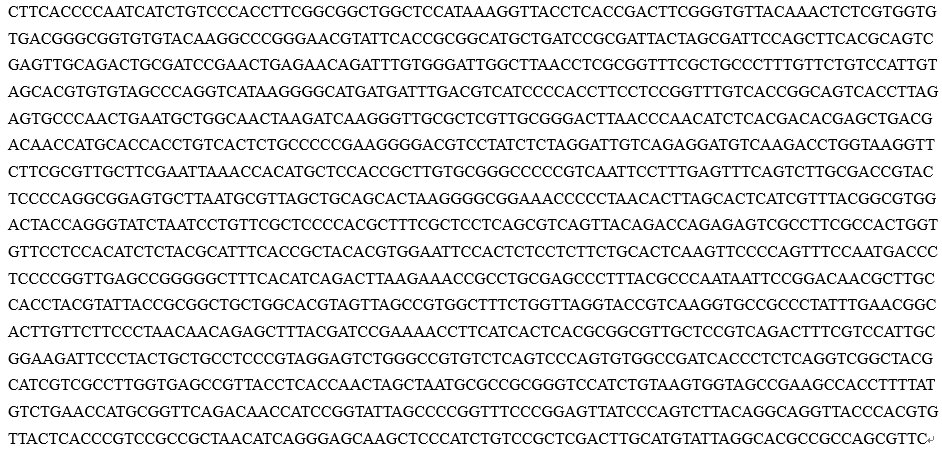


**Supplementary Fig. S3**

Sparse curve analysis was performed for control group (C) and treatment group (T).


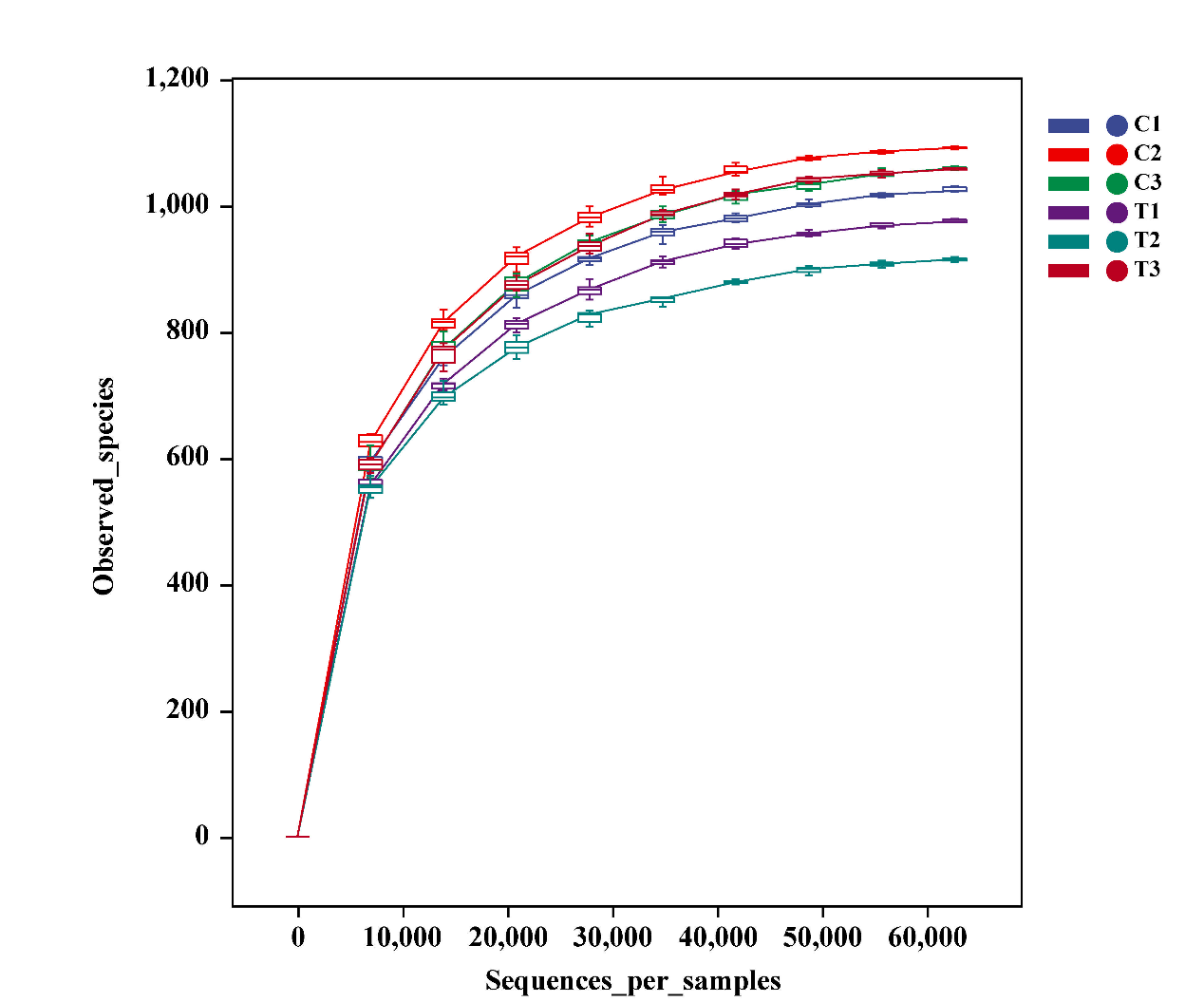

Supplement: Supplemental material — Tables S1 to S3; Fig. S1 to S3. [file spectrum.03995-25-s0001.docx]
